# LASIK-induced corneal changes after correction of hyperopia with and without application of Mitomycin-C

**DOI:** 10.1186/s12886-019-1100-7

**Published:** 2019-04-23

**Authors:** Ehab M. Moawad, Ahmed A. Abd Elghany, Amr A. Gab-Alla, Osama M. Elbassiouny, Mohsen S. Badawy

**Affiliations:** Department of ophthalmology, Faculty of Medicine Suez Canal University, Ismailia, Egypt

**Keywords:** Hyperopic LASIK - regression, Mitomycin-C

## Abstract

**Background:**

The study aimed to assess the role of intraoperative mitomycin-C (MMC) application during hyperopic LASIK correction (+ 1.00 D to + 6.00 D) by examining topographic corneal changes and incidence of regression over a one-year follow-up period.

**Methods:**

This comparative randomized control study included 68 hyperopic patients (136 eyes) divided into two groups; Group A included 34 patients (68 eyes) that had LASIK with the application of 0.02% MMC for 10 s on the stromal bed after excimer laser treatment, and group B included 34 patients (68 eyes) that had LASIK without MMC application. Uncorrected distance visual acuity (UDVA), refraction, keratometry and topography were recorded at 1st week and 1st, 3rd, 6th, and 12th months postoperation. Predictability and treatment efficacy were also recorded at the end of the follow-up period.

**Results:**

Better predictability was noted in group A than in group B at the 6 month and 12 month follow-up visits, with a mean cycloplegic refraction SE of + 0.5 ± 0.31 D in group A and + 0.67 ± 0.39 D in group B at the 6 month visit, and + 0.63 ± 0.37 D in group A and + 0.89 ± 0.48 D in group B at the 12 month visit. The efficacy of the treatment at the end of the follow up period was better in group A than in group B. Group A showed fewer topographic corneal changes than group B.

**Conclusions:**

Intraoperative MMC application during hyperopic LASIK achieves better predictability and efficacy and induces fewer topographic changes and lower regression rate of hyperopia during the first postoperative year.

**Trial registration:**

the Pan African Clinical Trial Registry PACTR201901543722087, on 29 January 2019.

## Background

Refractive surgical hyperopic correction represents a challenge for the refractive surgeon due to its unpredictability and high incidence of refractive regression [1]. The refractive surgical management of hyperopia has not gained the widespread acceptance and popularity among patients or physicians that surgical management for myopia has achieved [2].

Postoperative regression is a common issue after hyperopic-LASIK mostly due to postoperative epithelial, sub-epithelial and/or stromal hyperplasia. It is not yet clear which of these layers plays a prominent role in this complication [3].

Mitomycin-C (MMC) is a potent mitotic inhibitor that effectively blocks keratocyte activation and proliferation, it also play a role in preventing epithelial hyperplasia. The role of MMC has been evaluated in hyperopic femtosecond laser-assisted corneal surgery. Less regression was noted in hyperopic eyes treated with MMC 15 months after surgery, and better uncorrected distance visual acuity (UDVA) and less remaining sphere were observed, compared with non MMC-treated eyes. Furthermore, better predictability was noted with the use of MMC than when MMC was not applied [4].

This study aimed to assess the role of intraoperative MMC application during LASIK correction of hyperopia regarding topographic changes of the cornea and incidence of regression over a one-year follow-up.

## Methods

This comparative randomized control study included 68 hyperopic patients (136 eyes) coming to the Ophthalmology Clinic at Suez Canal University Hospital seeking laser refractive surgical correction. *Inclusion criteria* included patients above 21 years of age with hyperopia (SE ranging from + 1.00 D to + 6.00 D) with no contraindications for LASIK. *exclusion criteria* included patients with systemic diseases that affect refractive stability, e.g., uncontrolled diabetes; patients with systemic conditions that affect wound healing, e.g., rheumatoid arthritis; patients with any other ocular pathology e.g. keratoconus; patients with previous refractive corneal surgeries; patients with expected residual stromal bed after LASIK < 300 μm or target K > 48 D; patients with postoperative under/over correction (> ±0.5 D) or patients who could not fulfil one-year follow-up.

Patients who fulfilled the inclusion criteria were randomly divided into two groups. Group A included patients who underwent LASIK correction with the application of 0.02% MMC for 10 s on the stromal bed after excimer laser treatment, and group B included patients who underwent LASIK correction without application of MMC. In each group, patients were categorized as low to moderate hyperopia (SE + 1.00 to + 3.00 D) and high hyperopia (SE > + 3.00 to + 6.00D). All operations were performed by the same surgeon.

Preoperative assessment included CDVA, cycloplegic refraction analysis, keratometry and Pentacam (CSO, SIRIUS, Italy) analysis. All patients were followed up for 1 year after the primary procedure. Within this time frame, the patients were scheduled for follow up visits at 1 day,1 week, 1 month, 3 months, 6 months and 12 months postoperation for assessment by UDVA, cycloplegic refraction analysis, keratometry and analysis of the mean corneal thickness at the 6-mm optical zone by Pentacam.

Surgical procedures were conducted using Moria 2 microkeratomes (Moria, Antony, France) to create the corneal flaps. Superiorly hinged corneal flaps were created using a suction ring and 90- or 130-μm microkeratome depth plates according to the corneal thickness. The Schwind Amaris-500 E LASIK machine was used to perform the corneal stromal ablation and a 6.0-mm optical zone (with a peripheral transition zone of 9 mm) was programmed in all cases. In group A, we applied 0.02% MMC on the stromal bed for 10 s after laser ablation and then washed it by irrigation with balanced salt solution (BSS) for 20 s.

The association between variables (UDVA, refraction, keratometry and topography) was calculated using the “χ2 test” for comparison of the proportions and using the “t test” for comparison of normally distributed variables and “Mann-Whitney U test” for comparison of non-parametric variables between the two groups, with 95% confidence level or *p* value < 0.05, using Statistical Package for Social Science (SPSS) version 2015.

## Results

This study involved 33 male (49%) and 35 female (51%) patients. Group A included 34 patients (68 eyes), 15 (44.1%) were male patients and 19 (55.9%) were female patients. Group B included 34 patients (68 eyes), 18 (52.9%) were male patients and 16 (47.1%) were female patients.

The mean age of the study populations was 35.7 ± 11.3 years of age for group A and 34 ± 10.7 years of age for group B.

The preoperative CDVA was 0.96 ± 0.08 in group A and 0.95 ± 0.07 in group B. The refraction was + 3.2 ± 1.1 D in group A and + 3.3 ± 1 D in group B. Keratometry was 42 ± 1.5 D in group A and 41.6 ± 1.5 D in group B. The mean corneal thickness (at the 6-mm optical zone) was 553.8 ± 11.8 μm in group A and 551.3 ± 11.5 μm in group B.

Refractions at 6 months and the 12 months postoperation were higher in group B compared to group A. Keratometry values at the 12th month were Lower in group B than group A. Refraction and keratometry were assessed in follow-up visits, as shown at Table 1.

**Table 1:** Refraction and keratometry assessed during follow-up visits in the two study groups

	Group A (mean ± SD)	Group B (mean ± SD)	*P -*value
Refraction (D)			
Preoperative	+ 3.2 ± 1.2	+ 3.3 ± 1	0.627^1^
1 day postop.	+ 0.26 ± 0.21	+ 0.24 ± 0.22	0.609^2^
1 week postop.	+ 0.26 ± 0.21	+ 0.27 ± 0.19	0.739^2^
1 month postop.	+ 0.31 ± 0.21	+ 0.33 ± 0.22	0.526^2^
3 months postop.	+ 0.4 ± 0.24	+ 0.45 ± 0.27	0.305^2^
6 months postop.	+ 0.5 ± 0.31	+ 0.67 ± 0.39	**0.009** ^2*^
12 months postop.	+ 0.63 ± 0.37	+ 0.89 ± 0.48	**0.002** ^2*^
*p* –value	**< 0.001** ^3*^	**< 0.001** ^3*^	
Average keratometry (D)			
Preoperative	42 ± 1.5	41.6 ± 1.5	0.084^2^
1 day postop.	44 ± 2.9	44 ± 1.5	0.929^2^
1 week postop.	44.2 ± 1.5	44 ± 1.5	0.368^2^
1 month postop.	44.2 ± 1.5	43.9 ± 1.5	0.327^2^
3 months postop.	44.1 ± 1.5	43.9 ± 1.5	0.323^2^
6 months postop.	44 ± 1.5	43.7 ± 1.6	0.149^2^
12 months postop.	43.9 ± 1.5	43.6 ± 1.6	**0.038** ^2*^
*P –*value	**< 0.001** ^3*^	**< 0.001** ^3*^	

^1^Student’s *t* test; ^2^Mann-Whitney U test; ^3^Friedman test;

* Statistically significant at *p* <  0.05

**Fig. 1:**
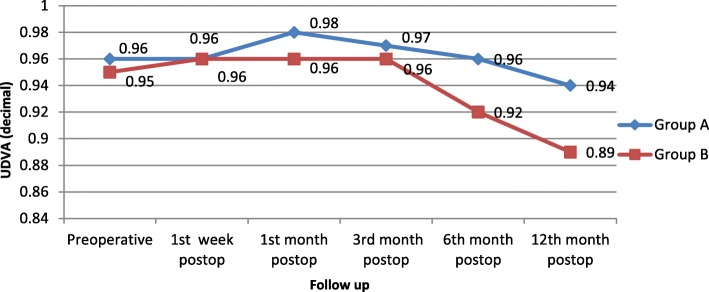
Change in UDVA (decimal) over 12 months postoperation in both study groups

Postoperative UDVA was assessed during follow-up and was found to decrease more in group B than group A, as shown in Fig. 1.

The mean corneal thickness at the 6-mm optical zone was measured at 1 week and 12 months postoperation, there was greater increase in the corneal thickness of the ablated zone (6-mm optical zone) in group B (11.4 ± 7.2 μm) than in group A (7.2 ± 4.6 μm) Table 2.

**Table 2:** Mean corneal thickness at the 6–mm optical zone preoperation, at 1 week postoperation and 12 months postoperation in the two study groups

Mean corneal thickness at the 6-mm optical zone (μm)	Group A (mean ± SD)	Group B (mean ± SD)	*p-*value
Preoperative	553.8 ± 11.8	551.3 ± 11.5	0.067^1^
1 week postop.	513.4 ± 16.2	506 ± 15.4	**0.0021** ^*^
12 months postop.	520.6 ± 15.4	517.4 ± 13.9	0.150^1*^
*P -*value	**< 0.001** ^2*^	**< 0.001** ^2*^	

^1^Student’s *t* test; ^2^Mann-Whitney U test; ^3^Friedman test;

*Statistically significant at *p* < 0.05

**Fig. 2:**
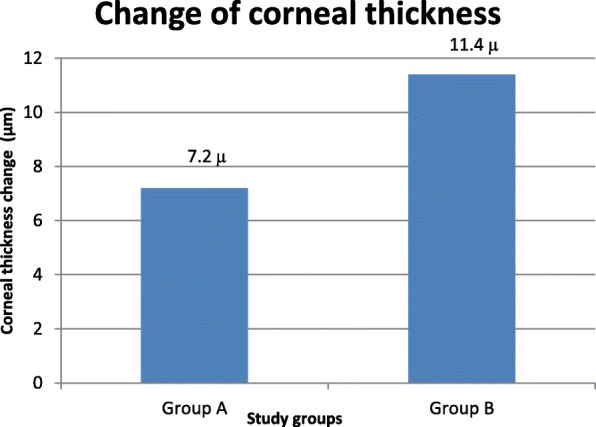
Change in corneal thickness (μm) over 12 months postoperation in both study groups was measured by the difference in the mean corneal thickness at the 6–mm optical zone at 12 months and 1 week postoperation

**Fig. 3:**
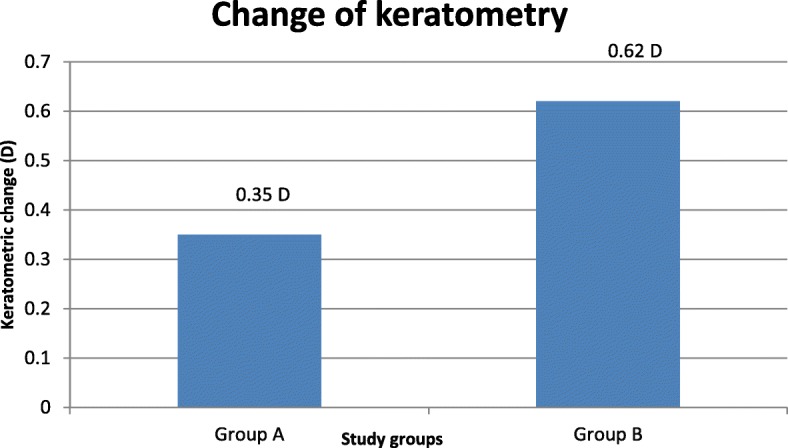
Change in keratometry (D) over 12 months postoperation in both study groups was measured by the difference in keratometry at 12 months and 1 week postoperation

Changes in corneal topography were assessed at follow-up by the difference in the mean corneal thickness at the 6-mm optical zone and by keratometry at 12 months and 1 week postoperation in the two study groups. There were fewer topographic changes at the first postoperative year in group A than group B, there was greater increase in the corneal thickness of the ablated zone (6-mm optical zone) in group B than in group A. The mean change in keratometry was lower in group A (− 0.35 ± 0.3 D) than in group B (− 0.56 ± 0.57 D) Figs. 2 & 3.

Regression of the refraction was assessed by the divergence of cycloplegic refraction from the intended level of correction (SE ≤ ±0.5 D) of more than + 0.5 D. There were 11 eyes (16.2%) showed regression of + 0.5 to + 1.00 D at the 12 month follow-up in group A compared to 20 eyes (29.4%) in group B, while only 3 eyes (4.4%) showed regression of more than + 1.00 D in group A compared to 8 eyes (11.8%) in group B Table 3.

**Table 3:** Regression of refraction in the two study groups

Regression	Group A (*n* = 68)	Group B(*n* = 68)	*P -*value
Regression of + 0.5 to + 1 D	11 (16.2%)	20 (29.4%)	**0.030** ^1*^
Regression of > + 1 D	3 (4.4%)	8 (11.8%)	
Mean ± SD	0.37 ± 0.34 D	0.61 ± 0.39 D	**< 0.001** ^2*^

^1^χ2 test; ^2^Mann-Whitney U test;

*Statistically significant at *p* < 0.05

**Fig. 4:**
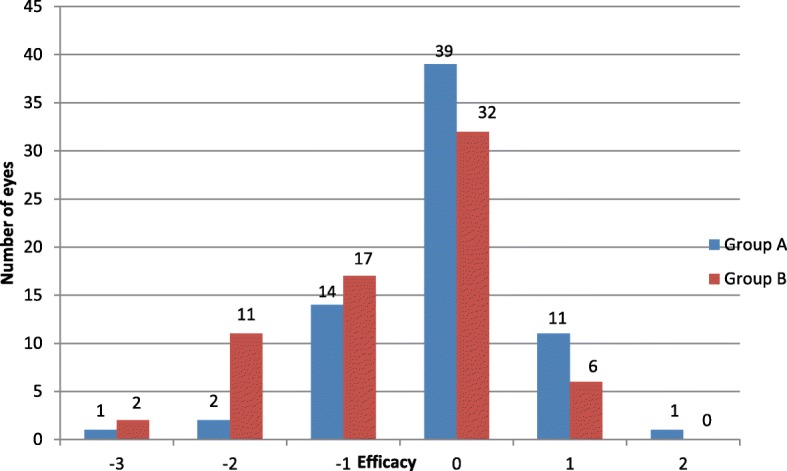
Treatment efficacy in the two study groups was assessed by number of lines of decimal gained or lost at the end over 12 months postoperation

The treatment efficacy assessed during follow-up in each group is shown in Fig. 4. A total of 39 eyes (57.4%) in group A and 32 eyes (47.1%) in group B showed post-operative UDVA at 12 months equal to preoperative CDVA. Eleven eyes (16.2%) gained 1 line of decimal in group A compared to 6 (8.8%) in group B. One eye (1.5%) gained 2 lines of decimal in group A. Fourteen eyes (20.6%) lost 1 line of decimal in group A compared to 17 eyes (25%) in group B, two eyes (2.9%) lost 2 lines of decimal in group A compared to 11 eyes (16.2%) in group B, and one eye (1.5%) lost 3 lines of decimal in group A compared to two eyes (2.9%) in group B.

**Fig. 5:**
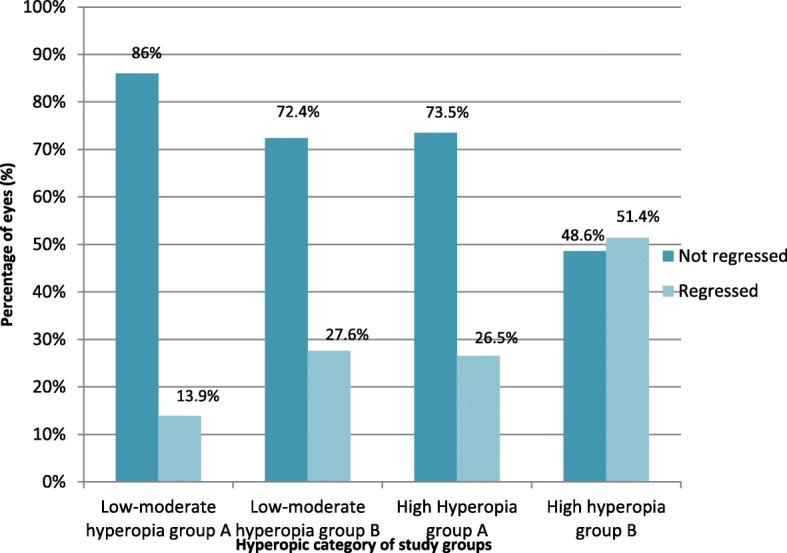
Regression in each subgroup regarding the amount of hyperopic correction

The incidence of regression was assessed in the two groups regarding the amount of hyperopic correction. In high hyperopic correction, regression was lower in group A than group B at 12 months postoperation. However, in low-moderate hyperopic correction there was no difference between the two groups at 12 months postoperation Fig. 5.

**Fig. 6:**
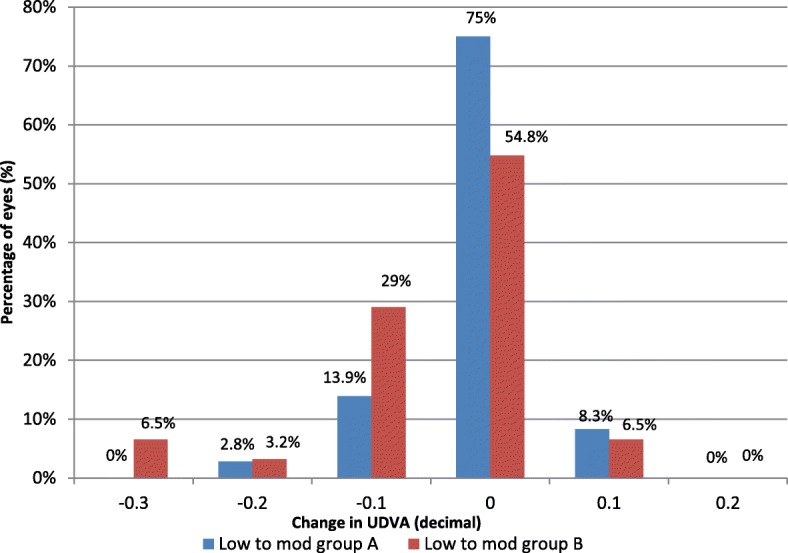
Treatment efficacy in the two low to moderate hyperopia subgroups was assessed by number of lines of decimal gained or lost at the end over 12 months postoperation

**Fig. 7:**
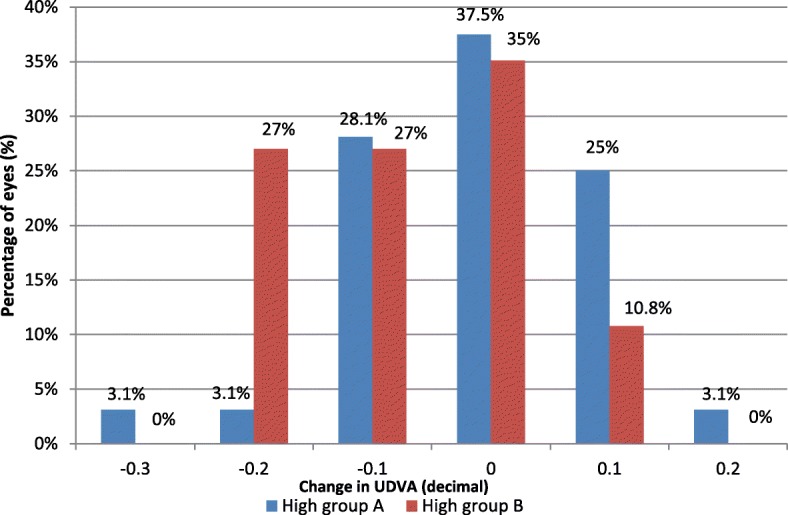
Treatment efficacy in the two high hyperopia subgroups was assessed by number of lines of decimal gained or lost at the end over 12 months postoperation

The treatment efficacy was also examined in both study groups regarding the amount of hyperopic correction as shown in Fig. 6 and Fig. 7. No intraoperative, early, or late postoperative complications were recorded.

## Discussion

This is the second study that involved the application of MMC in hyperopic laser correction. The first was by Garcia-Gonzalez M et al. [4], who found that eyes with hyperopia treated with MMC during FemtoLASIK showed better predictability and less regression than non-MMC treated eyes after 15 months of the primary procedure. Our study agreed with these results as we noticed better predictability in group A than in group B at the 6 month and the 12 month follow-up visits, with a mean cycloplegic refraction SE of + 0.5 ± 0.31 D in group A and + 0.67 ± 0.39 D in group B at the 6 month follow-up visit and + 0.63 ± 0.37 D in group A and + 0.89 ± 0.48 D in group B at the 12 month follow-up visit.

We also found better predictability at the 12 month follow-up visit for low to moderate hyperopia correction in both study groups (86% in ‘MMC treated’ group and 72.4% in ‘non-treat’ group were within ±0.5 D of intended correction) than for high hyperopia correction (73.5% in group A and 48.6% in group B were within ±0.5 D of the intended correction).

**Jaycock et al.** [5] also found that after 5 years of hyperopic LASIK correction, there was more acceptable predictability for the correction of low degrees of hyperopic refractive errors (+ 1.00 to + 3.00 D), with 22 of 31 eyes (71.0%) within ±1.00 D of the intended correction. Predictability for higher-order corrections (+ 3.5 to + 6.00 D) was less acceptable, with 6 of 16 eyes (37.5%) at ±1.00 D of the intended correction.

**Zadok et al.** [6] found better predictability in correction up to + 3.0 D (89% of eyes within ±1.0 D of emmetropia) with less predictability in laser correction of more than + 3.0 D (52% of eyes within ±1.0 D of emmetropia).

In the current study we also found less hyperopic regression at 12 months postoperation in group A than in group B, and 11 eyes (16.2%) showed regression of + 0.5 to + 1.00 D at the 12 month follow-up in group A compared to 20 eyes (29.4%) in group B, while only 3 eyes (4.4%) showed regression of more than + 1.00 D in group A compared to 8 eyes (11.8%) in group B.

**Garcia-Gonzalez M et al.** [4] also observed less regression at 3 months postoperation in hyperopic eyes treated with MMC than in non MMC-treated eyes. They found that 76.3% of eyes in the MMC-treated group were within ±0.50 D compared to 55.5% in the non-MMC-treated group, and 90.5 and 82.5% were within ±1.00D in the MMC-treated and non-MMC-treated groups, respectively. The incidence of retreatments during the 15 -month follow-up was significantly lower in the MMC-treated group than in the non-MMC-treated group (6.6% versus 10.5%, respectively).

In the current study, we found no significant difference in UDVA between the groups during the 3 months postoperation; however, UDVA was higher in ‘MMC treated’ group than in ‘non-treat’ group in follow up visits at 6 months (in ‘MMC treated’ group: 0.96 ± 0.08, ‘non-treat’ group: 0.92 ± 0.09) and 12 months (‘MMC treated’group: 0.94 ± 0.09, ‘non-treat’ group: 0.89 ± 0.11).

**Garcia-Gonzalez M et al**. [4] found a slight difference at 3 month postoperation: UDVA was 0.93 in the MMC-treated group and 0.87 in the non MMC-treated group, but no significant differences in UDVA were found at 15 months postoperation.

We agree with **Garcia-Gonzalez M et al**. [4] that the treatment efficacy was better in the MMC-treated group at the end of the follow-up period, but we noticed better efficacy at 12 months postoperation in the correction of low to moderate hyperopia than in high hyperopia in both groups.

To the best of our knowledge, we are the first to describe the relationship between hyperopic regression and topographic corneal changes. We assessed the postoperative topographic corneal changes in all patients by studying changes in the mean corneal thickness at the 6-mm optical zone and changes in keratometry using Pentacam at 1 week and at 12 months postoperation.

In the current study we found that there was greater increase in the corneal thickness of the ablated zone (6-mm optical zone) in ‘non-treat’ group (11.4 ± 7.2 μm) than in ‘MMC treated’ group (7.2 ± 4.6 μm). The mean change in keratometry was lower in in ‘MMC treated’ group (− 0.35 ± 0.3 D) than in ‘non-treat’ group (− 0.56 ± 0.57 D). This explains the lower regression in in ‘MMC treated’ group patients due to fewer topographic changes in the first postoperative year.

We also found that there were fewer topographic changes in patients who had not shown regression in each study group compared to patients who had shown regression. This also explains why hyperopic regression is related to topographic corneal changes, and we believe that MMC application prevents stromal and epithelial hyperplasia associated with the regression of hyperopia.

## Conclusion

Intraoperative MMC application during hyperopic LASIK achieves better predictability and efficacy and induces fewer topographic changes and regression during the first postoperative year especially for high hyperopic correction of more than + 3.00 D.

## Abbreviations

CDVA: Corrected distance visual acuity
MMC: Mitomycin-C
UDVA: Uncorrected distance visual acuity
